# Systematic Analysis of Small RNAs Associated with Human Mitochondria by Deep Sequencing: Detailed Analysis of Mitochondrial Associated miRNA

**DOI:** 10.1371/journal.pone.0044873

**Published:** 2012-09-11

**Authors:** Lakshmi Sripada, Dhanendra Tomar, Paresh Prajapati, Rochika Singh, Arun Kumar Singh, Rajesh Singh

**Affiliations:** 1 Department of Cell Biology, School of Biological Sciences and Biotechnology, Indian Institute of Advanced Research, Gandhinagar, India; 2 Department of Human Health and Disease, School of Biological Sciences and Biotechnology, Indian Institute of Advanced Research, Gandhinagar, India; French National Center for Scientific Research - Institut de biologie moléculaire et cellulaire, France

## Abstract

Mitochondria are one of the central regulators of many cellular processes beyond its well established role in energy metabolism. The inter-organellar crosstalk is critical for the optimal function of mitochondria. Many nuclear encoded proteins and RNA are imported to mitochondria. The translocation of small RNA (sRNA) including miRNA to mitochondria and other sub-cellular organelle is still not clear. We characterized here sRNA including miRNA associated with human mitochondria by cellular fractionation and deep sequencing approach. Mitochondria were purified from HEK293 and HeLa cells for RNA isolation. The sRNA library was generated and sequenced using Illumina system. The analysis showed the presence of unique population of sRNA associated with mitochondria including miRNA. Putative novel miRNAs were characterized from unannotated sRNA sequences. The study showed the association of 428 known, 196 putative novel miRNAs to mitochondria of HEK293 and 327 known, 13 putative novel miRNAs to mitochondria of HeLa cells. The alignment of sRNA to mitochondrial genome was also studied. The targets were analyzed using DAVID to classify them in unique networks using GO and KEGG tools. Analysis of identified targets showed that miRNA associated with mitochondria regulates critical cellular processes like RNA turnover, apoptosis, cell cycle and nucleotide metabolism. The six miRNAs (counts >1000) associated with mitochondria of both HEK293 and HeLa were validated by RT-qPCR. To our knowledge, this is the first systematic study demonstrating the associations of sRNA including miRNA with mitochondria that may regulate site-specific turnover of target mRNA important for mitochondrial related functions.

## Introduction

Mitochondria are known for a long time as power house of cell and are primary site for various metabolic activities like respiration, fatty and amino acid metabolism [1], [2], [3]. Increasing evidences suggest its role in many other processes like cell death, inflammation and innate immune response [4], [5], [6]. The role of mitochondria is now known to be associated with wide range of pathological conditions like cancer, neurodegeneration, ageing, inflammation and infection [7], [8], [9]. The further study of mitochondrial functions and its regulation in various pathological conditions needs to be elucidated.

Human mitochondrial genome is 16.5 kb circular DNA and is known to encode 13 proteins subunits of mitochondrial respiratory complexes, 22 tRNA and 2 rRNAs [10], [11]. The mitochondria are not completely autonomous and require nuclear encoded proteins for their optimal function. Interestingly, proteome of mitochondria from several species including human have been now well studied and is known to contain more than 1500 proteins [12]. The majority of proteins are encoded by nuclear DNA, translated in cytoplasm and imported to mitochondria. The translocation of proteins to different compartments of mitochondria is known to occur through TOM/TIM complexes [13], [14]. Similarly, many nuclear encoded RNAs are also known to be imported to mitochondria [15]. The translocation of tRNA to mitochondria is known for long time [16]. 5S rRNA is also translocated to mitochondria from cytosol with help of rhodanese and L18; assembles into mitochondrial ribosomes [17]. Nuclear encoded ncRNA components of RNase P and MRP (mitochondrial RNA processing) enzyme complexes are translocated to mitochondrial matrix to cleave polycistronic mRNA and initiate mitochondrial DNA replication respectively [18], [19]. The PNPASE (polynucleotide phosphorylase) complex involved in translocation of these RNAs has also been characterized [20]. These evidences suggest that there is an active transport of nuclear encoded RNAs to/in mitochondria.

Emerging evidences also suggest that the mitochondrial membrane serves as a novel platform to assemble RNA granule comprising of the proteins and RNA involved in RNA metabolism [21]. Mitochondria are also known to have dynamic interactions with P bodies [22], [23], nuage bodies [24] and stress granules [25]. These evidences suggest that mitochondrial membrane may anchor RNA binding proteins, which may bind to small RNAs (sRNAs) including miRNA. Recently, miRNAs have also been found in mitochondria isolated from different tissues and different organisms: 15 miRNAs in rat liver mitochondria [26], 20 miRNAs in mouse liver mitochondria [27], 20 miRNAs in mitochondria from human myotubes [28] and 13 miRNAs in mitochondria from HeLa [29]. However, the physical association of small non-coding RNA including miRNA with mitochondria has not been well studied. We planned to use deep sequencing method to systematically analyze the association of sRNA with mitochondria. The deep sequencing method of fractionated RNA has become one of the most powerful tools to discover novel sRNA [30]. This approach has been widely used in finding novel sRNA although it does not provide detailed information about their spatial or temporal regulation. Therefore, we planned to combine subcellular fractionation and deep sequencing method to identify sRNA specifically associated with mitochondria. The method revealed that sRNAs including miRNA, piRNA, tRNA, rRNA and repeat associated RNA are associated with mitochondria. We specifically focused on analysis of miRNA and found that mitochondria are one of the post-transcriptional destinations of miRNA.

## Results

### Isolation and Analysis of Mitochondrial RNA

**Figure 1:**
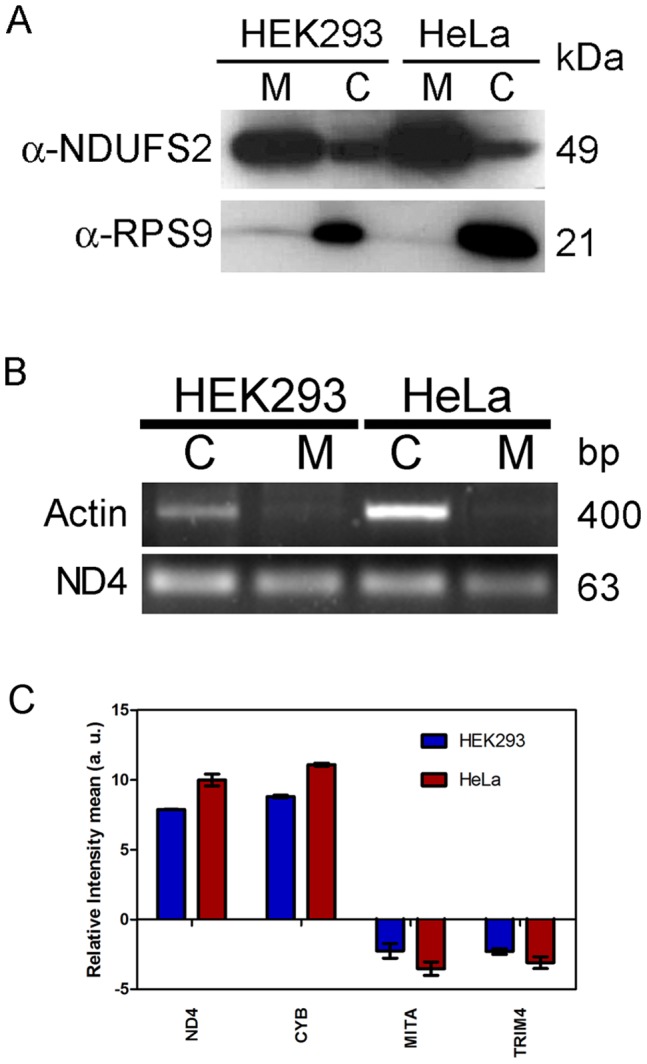
Analysis of isolated mitochondria. The mitochondria were isolated and purified from HEK293 and HeLa. (A) The protein contents of whole cell lysate, and purified mitochondria were normalized, resolved on 12.5% SDS-PAGE, transferred to PVDF membrane and probed with NDUFS2 and RPS9 antibody. (B) RNA was isolated from purified mitochondria and total cell. The subsequent cDNA was used for PCR amplification of mitochondrial encoded ND4 and cytosolic/nuclear specific β-actin. M: mitochondrial fraction; C: cellular lysate. (C) RNA was isolated from mitochondria. The nuclear RNA contamination in mitochondrial RNA was assessed by checking relative enrichment of mitochondrial encoded RNA (ND4, CYB) and nuclear encoded mRNA (TRIM4, MITA) by RT-qPCR as described in method section.

To analyze the sRNA families associated with mitochondria, a highly purified fraction of mitochondria was prepared from HeLa and HEK293 cell lines. The purity of mitochondrial fraction was confirmed by western blotting (Figure 1A). It was observed that NDUFS2, a subunit of mitochondrial complex-I, is highly enriched in purified mitochondrial fraction whereas RPS9 was only detectable in total cell and not in purified mitochondrial fraction. Ribosomal protein S9 (RPS9) is S4P family protein, a component of 40S ribosomal subunit, which is localized mainly in nucleolus and cytoplasmic ribonucleic component [31] but not in mitochondria. Hence, RPS9 was used to determine nuclear and cytosolic contamination. This evidence strongly suggests that purified mitochondrial fraction was free from nuclear as well as polysomal contamination.

RNA was isolated from total cells and purified mitochondria from both HEK293 and HeLa. The quality of RNA was determined by RNA integrity number (RIN values). The total cellular RNA showed clear peak of 28S and 18S RNA at 50 sec and 43 sec with RIN values of 8.5 and 10 for HEK293 and HeLa respectively. The microfluidic electrophoresis of mitochondrial RNA showed several peaks. It suggests presence of sRNAs other than 28S and 18S therefore traditional RIN may not be valid for mitochondrial RNA integrity. The purity of mitochondrial RNA was also analyzed at RNA level by RT-PCR of β-actin, nuclear encoded gene and ND4, mitochondrial encoded gene. The nuclear encoded mRNA, β-actin was only detected in the RNA from total cells and was absent in the mitochondrial fraction (Figure 1B) whereas ND4 was detected both in mitochondrial as well as total cellular RNA (HEK293 and HeLa) as expected. This suggests that there is no non specific association of nuclear encoded RNA with mitochondria. The mitochondrial RNA purity was further analyzed by qPCR for cytosolic RNA contamination by analyzing the presence of two nuclear encoded mRNA (TRIM4 and MITA) and two mitochondrial DNA encoded (ND4 and CYB) mRNA were taken as positive controls. The mitochondrial encoded mRNA was enriched significantly in mitochondrial fraction as compared to cytosolic mRNA (Figure 1C). These two experiments also suggest that mitochondrial RNA further analyzed by deep sequencing, is free from nonspecific association of nuclear encoded RNA.

### Analysis of sRNA Libraries Associated with Human Mitochondria

The sRNA (18–30 nucleotides (nt)) associated with mitochondria was isolated, library generated and sequenced using Illumina high-throughput sequencing platform. The sequencing generated 19580503 and 17743919 raw sequencing reads associated with mitochondria of HEK293 and HeLa respectively. The dataset was deposited into NCBI Gene Expression Omnibus [accession No: GSM797669 and GSM797670 for sRNA associated with mitochondria of HEK293 and HeLa respectively]. Screening for 3′ adaptor sequences and 5′ adaptor contaminations using a local blastn program resulted in the elimination of 1749595 and 66668 tags from HEK293 and HeLa respectively. The reads of mRNA degraded products with poly-A sequence were low (350 and 215 reads each in HEK293 and HeLa respectively) indicating that the libraries were devoid of degraded mRNA products. The length of 731292 and 471296 reads in HEK293 and HeLa respectively were smaller than 18 nt, hence removed leaving behind 19089819 and 17312962 sequences for annotation of HEK293 and HeLa respectively.

**Figure 2:**
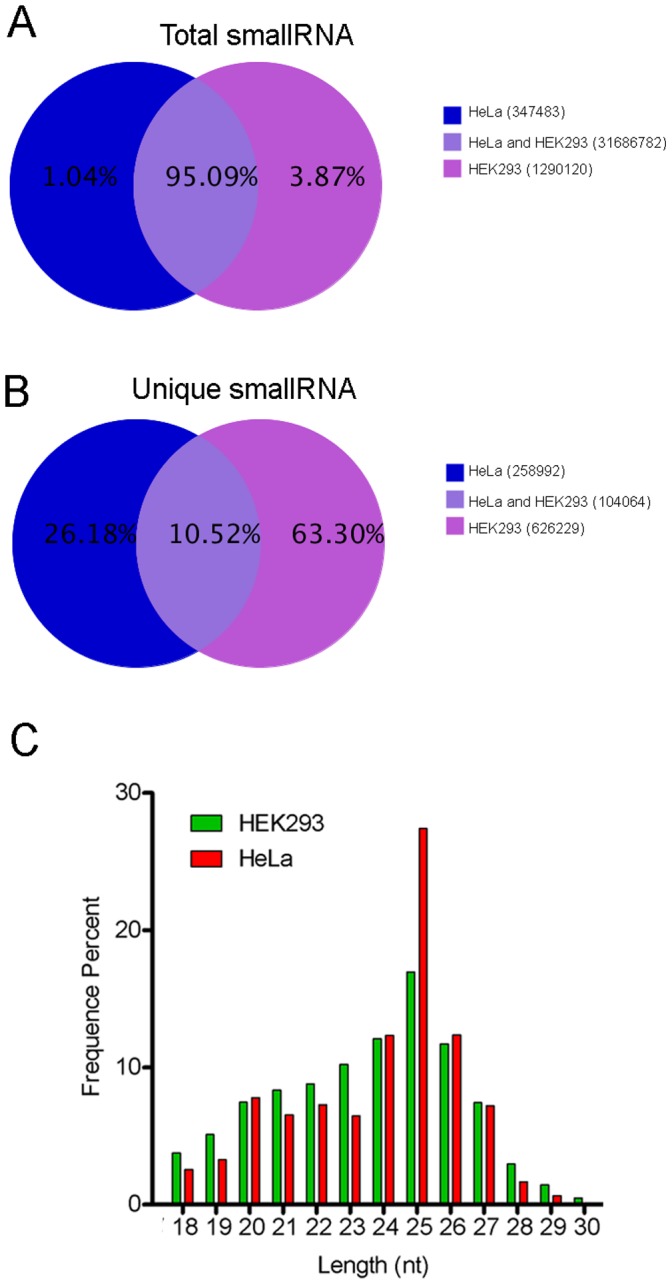
Generation and analysis of sRNA sequences from mitochondria. sRNA library generated from mitochondria from HEK293 and HeLa were sequenced using Illumina Hiseq 2000 platform that generated 19089819 and 17312962 clean sequence respectively. (A) Venn diagram showing distribution of common and specific sRNA total sequence reads amongst the two libraries. (B) Venn diagram showing distribution of common and specific sRNA unique sequence reads amongst the two libraries. (C) Length distribution and frequency percent of sequences in HEK293 and HeLa mitochondrial sRNA libraries.

The further analysis of sRNA library showed that 95% of total sequences (31686782 reads) corresponding to 10.52% unique sequence (104064 reads) were common in both HEK293 and HeLa. 3.87% total sequences (1290120 reads) corresponding to 63.3% unique sequences (626229 reads) and 1.04% total sequences (347483 reads) corresponding to 26.18% unique sequences (258992 reads) were specifically associated with mitochondria of HEK293 and HeLa respectively (Figure 2A, Figure 2B).

The length of miRNA, piRNA and siRNA are generally 21–22, 30 and 24 nt respectively [32]. Thus, the analysis of sRNA length helps to categorize the sRNA population obtained from both the cell lines. The length of 85.3% (14349816) and 91.2% (15070360) sequences from HEK293 and HeLa respectively were between 20–27 nt. The highest number of sequences were of 25 nt in length (17% and 27% sequences from HEK293 and HeLa respectively) (Figure 2C) indicating the abundance of sRNA other than miRNA and piRNA.

The sRNA sequences were mapped to human reference genome (UCSC hg19) to determine their origin and distribution. 87.58% (14728844 reads) and 92.67% (15297190 reads) from HEK293 and HeLa respectively aligned to the human genome in sense/antisense orientation. Majority of the sRNA reads aligned to the sense strand of uncharacterized region of genome (chrUn_g1000220) followed by antisense stands of chromosome 8 and 2 (Figure S1A, Figure S1B).

**Figure 3:**
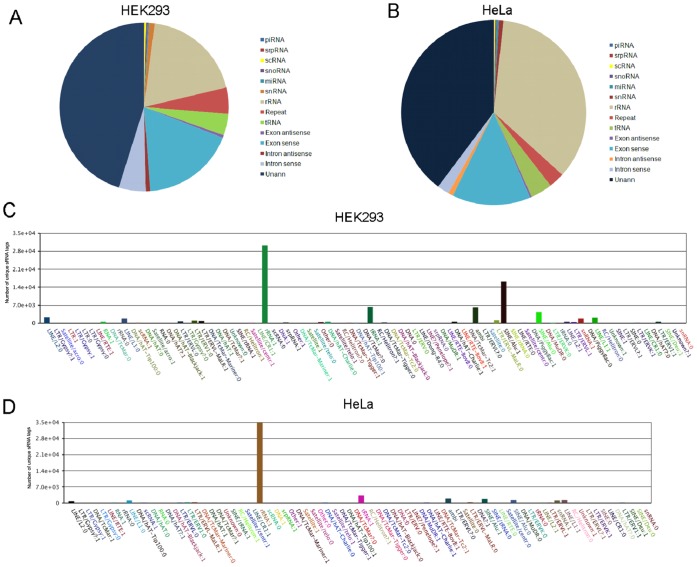
Frequency of distribution of different classes of RNA associated with mitochondrial sRNA libraries. The unique sequences obtained from the sRNA libraries were subjected to a series of sequence similarity searches using specific databases (rRNAs, tRNAs, snRNAs, snoRNAs, miRNAs, other non-coding RNAs). The sequences that did not match with any known sequence were considered as unannotated sequences. (A) An overview of sRNA associated with mitochondria of HEK293. (B) An overview of sRNA associated with the mitochondria of HeLa. The unique clean tags of repeat associated sequences were further categorized to determine the diversity of repeat associated RNA. (C) Detailed clustering of repeat-associated RNAs from mitochondria of HEK293. (D) Detailed clustering of repeat-associated RNAs from mitochondria of HeLa.

The sRNA sequences were annotated according to their overlap with sequences of known RNA in Genbank and Rfam (Figure 3A, Figure 3B, Table S1, Table S2). As expected, the most abundant sRNA classes in the both the libraries were rRNA (78.11% in HEK293 and 91.76% in HeLa), tRNA (3.96% in HEK293 and in 0.88% in HeLa) and unannotated sRNA (8.47% in HEK293 and in 3.27% in HeLa). The sequences corresponding to exons/introns were low (1.21% in HEK293 and in 0.11% in HeLa) suggesting the libraries were devoid of degraded mRNA products. The other categories of sRNA included snRNA (1.21% in HEK293 and 0.11% in HeLa), piRNA (0.01% in HEK293 and HeLa), srpRNA (0.17% in HEK293 and 0.01% in HeLa), snoRNA (0.21% and 0.03% in HEK293 and HeLa respectively) and repeat associated sRNAs. The top five abundant classes of repeats observed from mitochondria of both cell lines were rRNA (47.32% and 99.26% HEK293 and HeLa respectively), tRNA (1.63% and 0.36% HEK293 and HeLa respectively), LINES (0.104%and 0.057% HEK293 and HeLa respectively), SINE/Alu (0.15% and 0.028% HEK293 and HeLa respectively) and snRNA (0.32% and 0.072% HEK293 and HeLa respectively). Low levels of LTR/ERVL (0.015% and 0.007% HEK293 and HeLa respectively), LTR/ERV1 (0.015% and 0.007% HEK293 and HeLa respectively) and LTR/ERVL-MaLR (0.015%1 and 0.006% HEK293 and HeLa respectively) were also observed (Figure 3C, Figure 3D, Figure S2A, Figure S2B).

### Analysis of known miRNAs Associated with Mitochondria

Our major interest was to investigate the association of miRNA with mitochondria hence we focused our analysis on miRNA. A total of 2249 unique tags (4.21% or 710742 sequence reads) and 1584 unique tags (2.58% or 426907 sequence reads) associated with mitochondria of HEK293 and HeLa respectively were categorized as miRNA (Table 1). The counts of miRNA varied from 1 to more than 100000. 428 and 327 mature miRNAs from HEK293 and HeLa respectively were observed to be associated with mitochondria. According to miRBase 17.0, we also found the association of 65 and 60 miRNA* with mitochondria of HEK293 and HeLa respectively.

**Table 1:** Summary of known miRNAs from mitochondrial sRNA libraries.

	miRNA	miRNA*	unique sRNA matched to miRNA^1^	total sRNA matched to miRNA^2^
**Known miRNA in miRBase**	1539	194	–	–
**HEK293**	428	65	2249	710742
**HeLa**	327	60	1584	426907

The clean sequence reads aligned to miRBase 17.0 to determine diversity and occurrence of miRNA in sRNA library.

1 count of unique sRNA sequences that matched to miRBase,

2 count of total sRNA sequences that matched to miRBase in HEK293 and HeLa.

### Analysis of Differential Association of miRNAs with Mitochondria of HEK293 and HeLa

**Figure 4:**
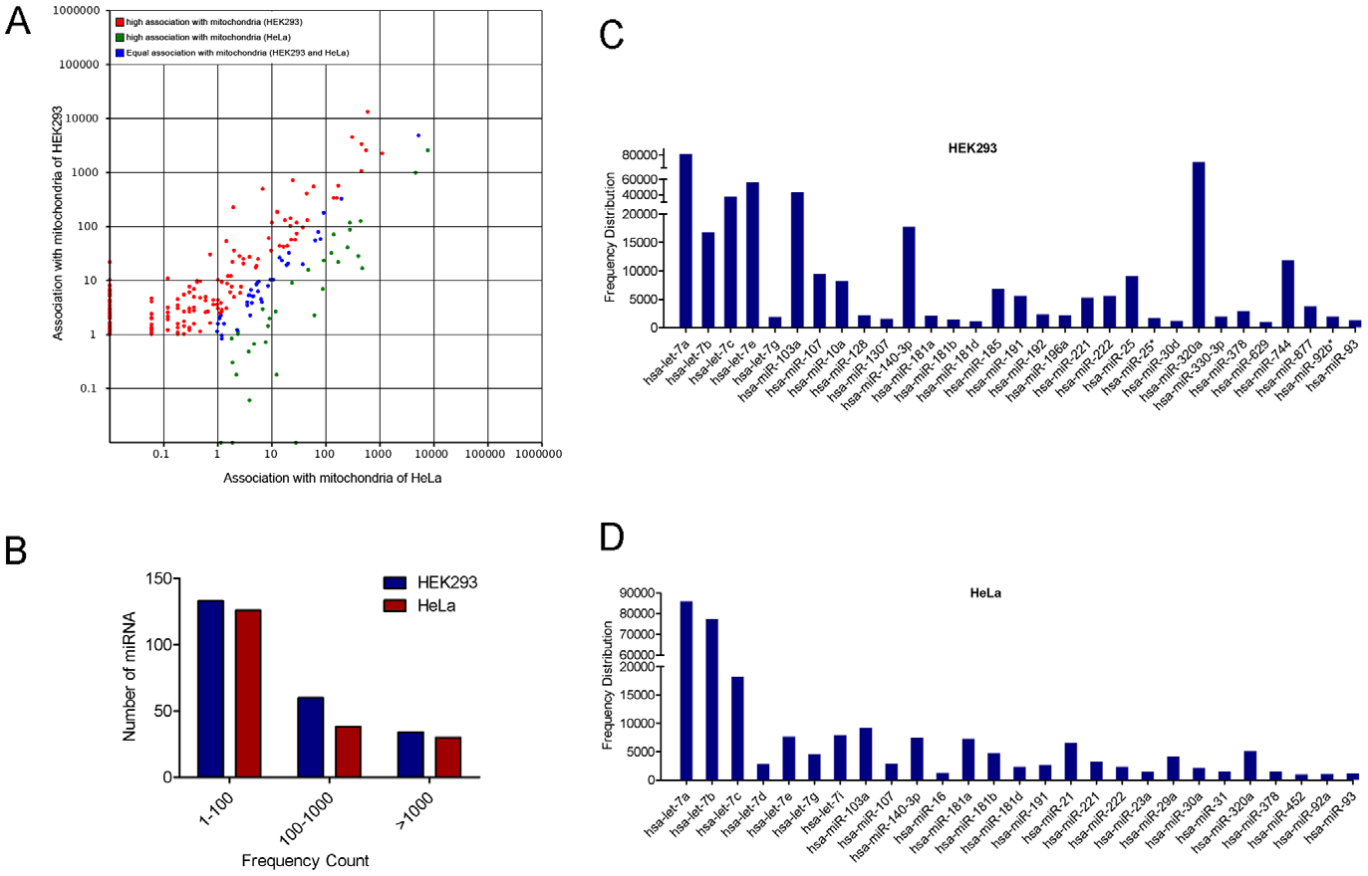
Analysis of differential association of miRNAs to mitochondria from HEK293 and HeLa. (A) Scatter Plot depicting the differential association of miRNAs from libraries of HeLa and HEK293. The X and Y axis shows association level of miRNAs with mitochondria from two cell lines. Red points represent miRNA with ratio>2; Blue points represent miRNA with 1/2<ratio<2; Green points represent miRNA with ratio<1/2. Ratio = normalized association of the HEK293/HeLa. (B) Distribution of known miRNAs: Numbers of sequence reads are taken as miRNA levels and the values are represented in the form of range of values in both HEK293 and HeLa sRNA libraries. (C) The frequency of highly associated miRNAs (>10,000 counts) with mitochondria of HEK293. (D) The frequency of highly associated miRNAs (>10,000 counts) with mitochondria of HeLa.

The differential association of miRNA to mitochondria of both HEK293 and HeLa has been summarized in Table S3. The frequency counts of 35 miRNAs associated with mitochondria of both the cell lines was similar (Figure 4A). The frequency count of miRNA ranged from 1 to more than 100,000 (Figure 4B). The most abundant miRNAs associated with mitochondria of HEK293 and HeLa were hsa-miR-423-5p, hsa-miR-320a and let-7 family members (let-7a, let-7b, let-7c, let-7d, let-7e, let-7f, let-7g, let-7h and let-7i) followed by hsa-miR-103b, hsa-miR-140-3p, hsa-miR-744, hsa-miR-107 (Figure 4C, Figure 4D, Table S3). hsa-miR-10a, hsa-miR-128, hsa-miR-1307, hsa-miR-140-3p, hsa-miR-185, hsa-miR-196a, hsa-miR-25, hsa-miR-320a, hsa-miR-330-3p, hsa-miR-340, hsa-miR-423-5p, hsa-miR-629 and hsa-miR-744 significantly associated with the mitochondria of HEK293. Similarly let-7i, hsa-miR-181b, hsa-miR-21, hsa-miR-23a, hsa-miR-29a, hsa-miR-30a, hsa-miR-31 and hsa-miR-452 associated significantly with mitochondria of HeLa.

### Analysis of Putative Novel miRNAs Associated with Human Mitochondria

**Figure 5:**
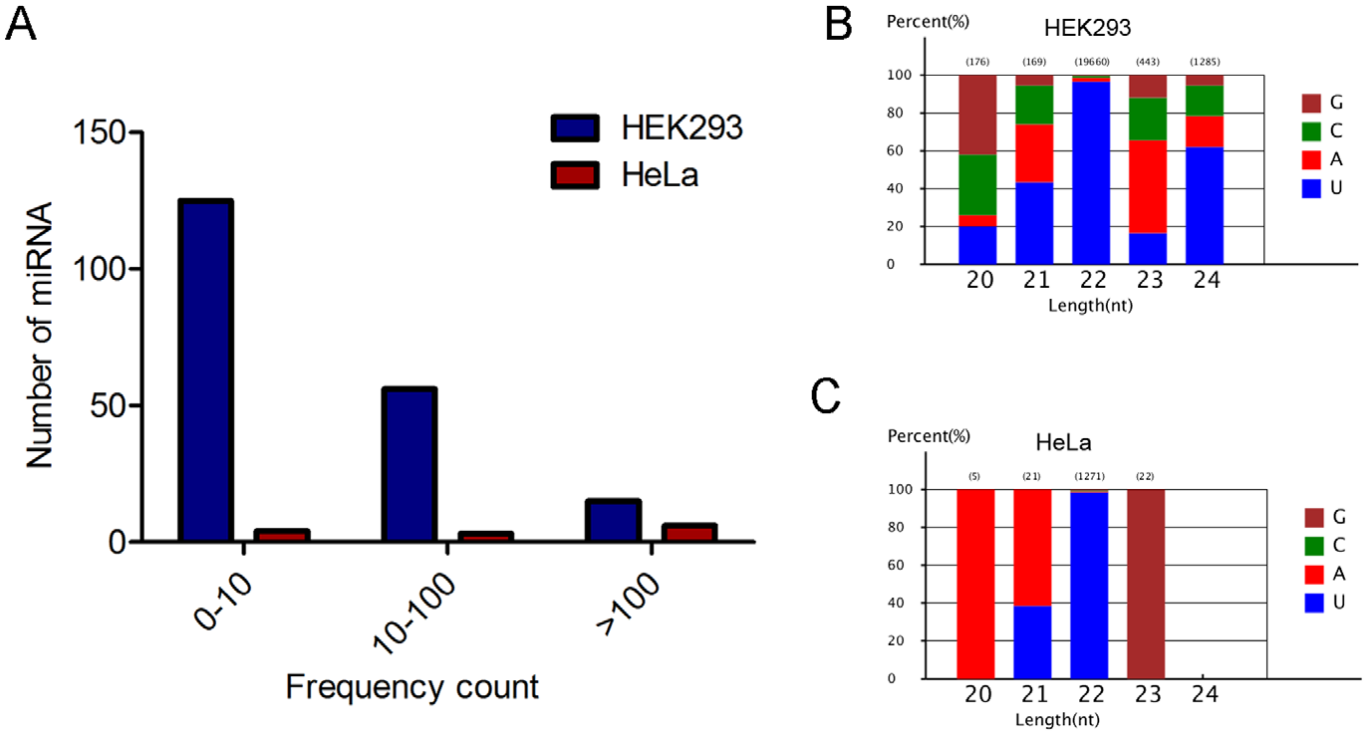
Analysis of putative novel miRNAs associated with human mitochondria. The putative novel miRNAs were predicted from unannotated clean reads using Mireap software. (A) The distribution of putative novel miRNAs levels with respect to frequency. Numbers of sequence reads are taken as miRNA levels and the values are represented in the form of range of values in both HEK293 and HeLa mitochondrial sRNA libraries. (B) The percentage of first nucleotide base bias of 18–23 nt putative novel miRNAs from mitochondria-associated sRNA library of HEK293. (C) The percentage of first nucleotide base bias of 18–23 nt putative novel miRNAs from mitochondria-associated sRNA library of HeLa.

As described above we observed significant number of reads from unannotated region of chromosome. The unannotated sequences were analyzed through computational pipeline described in methods to classify them as putative novel miRNA. In total, 196 and 13 putative novel miRNAs were identified by virtue of their ability to form miRNA-like single hairpins and other criteria of miRNA from the mitochondrial sRNA libraries of HEK293 and HeLa respectively (Table S4). Here, we used the naming convention ‘‘293m8-miRX’’ and ‘‘HM3-miRX’’ for each putative novel miRNAs associated with mitochondria of HEK293 and HeLa respectively. The counts of putative novel miRNAs ranged from 5–3132 in HEK293 and 5–208 in HeLa (Figure 5A). Interestingly, some putative novel miRNAs had similar sequence, count and structure but mapped to different genomic locations. Analysis of the first nucleotide bias of the 18–30 nt miRNA candidates revealed that uridine (U) was the most common base (>90%) at positions 1, 19, 20, 21, 22 of 22 nt long putative novel miRNAs (data not shown). The analysis of 20–24 nt miRNAs showed that the first nucleotide of 40% of 20 nt long miRNAs started with G, 40% of 21 nt long miRNAs with U, >90% of 22nt long miRNAs with U, >50% of 23 nt long miRNAs with A and >60% of 24 nt long miRNAs with U in HEK293 (Figure 5B). Interestingly, in HeLa 100% of 20 nt long miRNAs began with A, 60% of 21 nt long miRNAs with A, 40% of 21 nt long miRNAs with U, >95% of 22 long miRNAs with U and 100% of 23 nt long miRNAs with G (Figure 5C).

### Alignment of sRNAs to Human Mitochondrial Genome

**Figure 6:**
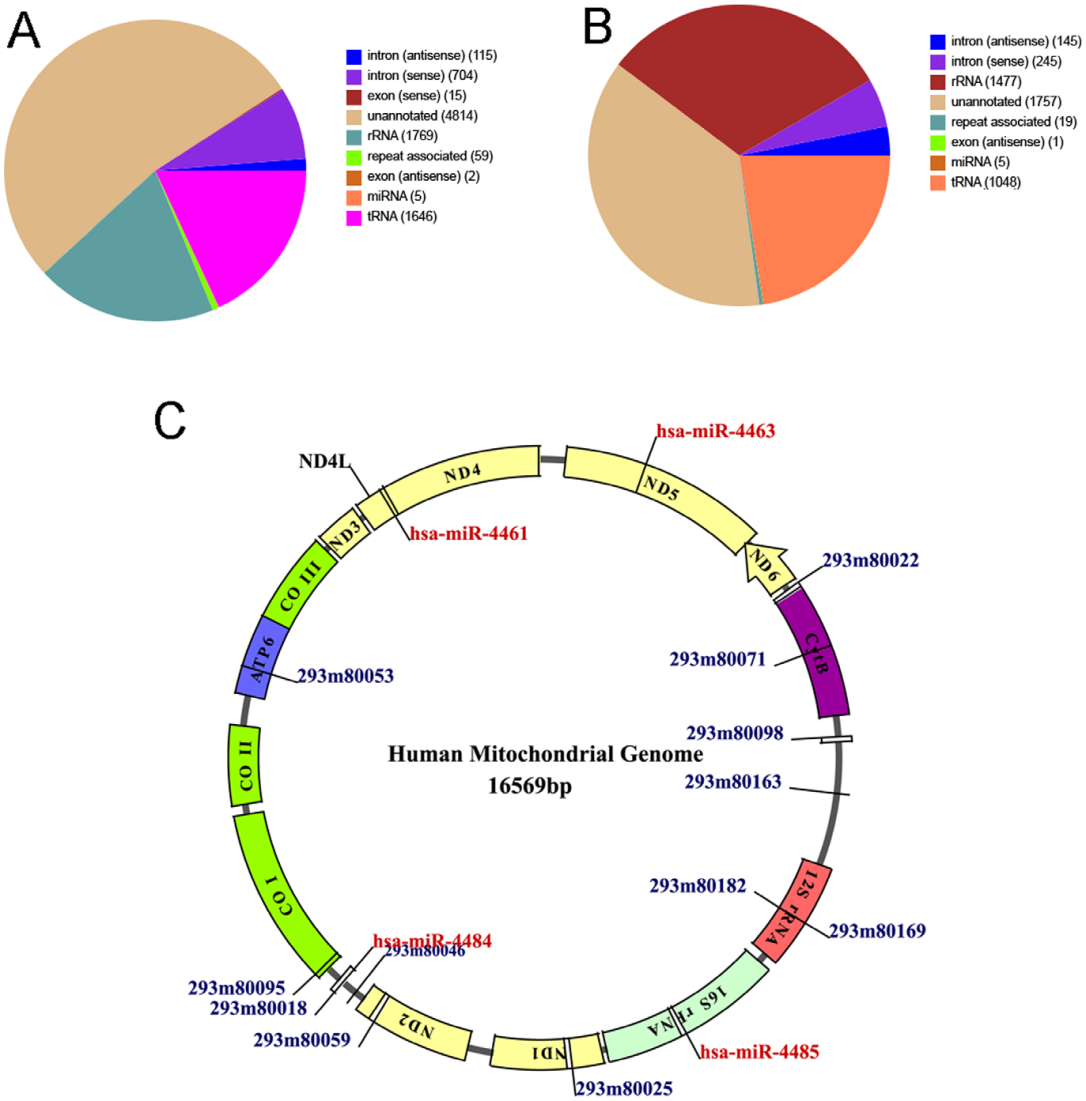
Mapping sRNAs to human mitochondrial genome. The sequences obtained from the mitochondrial sRNA libraries from HEK293 and HeLa were aligned to mitochondrial genome. (A) An overview of mitochondria-associated sRNAs from HEK293 that aligned to mitochondrial genome. (B) An overview of mitochondria-associated sRNAs from HeLa that aligned to mitochondrial genome. (C) The miRNAs and putative novel miRNAs that aligned to mtDNA (determined by SOAP, MapMi, BLASTN and RNAhybrid) were mapped on mtDNA using Dynamo Software tool. The locations of known miRNAs and putative novel miRNAs that aligned to mtDNA are marked in red and blue respectively. If more than 1 miRNA aligned to same position, only 1 miRNA was marked.

Human mitochondria contain 16.5 kb circular DNA as its own genome. We further studied the alignment of sRNAs with mitochondrial genome. We used two different approaches to search for the sequences that aligned to mitochondrial genome. Firstly, SOAP parameters described in method section, showed that 4697 unique sequences (corresponding to 14102 reads) from HEK293 and 9129 unique sequences (corresponding to 78374 reads) from HeLa libraries aligned to human mitochondrial genome. The aligned sequences belonged to unannotated (40.9% in HEK293 and 16.8% in HeLa), tRNA (38.2% in HEK293 and 40.1% in HeLa) and rRNA (16.4% in HEK293 and 39.4% in HeLa) categories. Some sequences were also categorized as repeat associated sequences (0.36% in HEK293 and 0.14% in HeLa) and miRNA (0.01% in HEK293 and 0.035% in HeLa) (Figure 6A, Figure 6B). The analysis showed that only four miRNAs: hsa-miR-4461, hsa-miR-4463, hsa-miR-4484 and hsa-miR-4485 aligned to mitochondrial genome at positions (10690–10712), (13050–13068), (5749–5766) and (2562–2582) corresponding to ND4L, ND5, L-ORF and 16S rRNA genes respectively. Analysis using another tool MapMi version 1.0.4.02 [33] resulted in alignment of 744 unique sequences to mitochondrial DNA. We also used local BLASTN version 2.2.20 program to screen putative novel miRNA alignments. We found the alignment of first 11 bases of 24 putative novel miRNAs at different positions of mitochondrial genome (Figure 6C). The 7 putative novel miRNAs aligned to non coding region, 5 to ATP6, 3 to tRNA, 2 to 12S rRNA, 2 to ND2 and 1 to HVRI, COI, CytB and ND1 gene region.

### Biological Processes Regulated by miRNAs Associated with Mitochondria

An individual miRNA may regulate several mRNAs in a pathway thus fine-tuning the cellular processes. Majority of target prediction tools give many false positive results therefore it has to be validated by both computational and experimental tools. We used Starbase that has been developed based on recent studies of CLIP (Cross-linking immunoprecipitation) and RNA degradome sequencing experiments (high-throughput sequencing data from 21 CLIP-Seq and 10 Degradome-Seq experiments from six organisms) [34]. The false positives targets were further eliminated by taking intersections of targets from three prediction tools. Therefore, the outcome obtained from this analysis may result in reliable targets mimicking closely to the physiological conditions.

Identified miRNAs were divided in two subgroups on the basis of frequency of association with mitochondria from HEK293 and HeLa: highly abundant miRNAs (count >5000) and less abundant miRNAs (count <5000). The targets of these miRNAs were further analyzed using DAVID to functionally annotate the identified targets into smaller and biologically meaningful groups. The targets of highly abundant miRNAs populated many GO categories with significant enrichment (>1.0) and p-Value (<0.05). GO terms related to regulation of transcription (GO: 0045449, GO: 0010629) were significantly enriched. The other significantly enriched categories included GO: 0051301∼cell division, GO: 0007049∼cell cycle, GO: 0016568∼chromatin modification, GO: 0035195∼gene silencing by miRNA, GO: 0007389∼pattern specification process, GO: 0001701∼in utero embryonic development, GO: 0042921∼glucocorticoid receptor signaling pathway (Table S5). To analyze the role that miRNAs play in the regulatory networks, we assigned putative miRNA targets into KEGG pathways, and observed that ubiquitin mediated proteolysis pathway was significantly enriched (p-value <0.05) (Table S6).

The GO analysis of the targets of less abundant miRNAs associated with mitochondria (<5000) showed significant enrichment of GO terms related to regulation of transcription (GO: 0045449, GO: 0010629, GO: 0006357, GO: 0051252) and the regulation of biosynthetic processes (GO: 0031327/28, GO: 0009891) (Table S7). To further analyze the networks we also used KEGG and found that pathways in cancer (solid tumors) and chronic myeloid leukemia were significantly enriched (p<0.05). Similarly TGF, Wnt and cell cycle pathway genes were also found be to significantly enriched (p<0.05) (Table S8).

The targets of putative novel miRNAs associated with mitochondria of HEK293 showed enrichment for positive/negative regulation of transcription (GO: 0006350∼transcription, GO: 0051252∼ RNA metabolic process, GO: 0010629∼negative regulation of gene expression). The nucleic acid, protein metabolic and catabolic related GO terms were also found to be enriched (GO: 0051254∼positive regulation of RNA metabolic process, GO: 0045935∼positive regulation of nucleic acid metabolism, GO: 0030163∼protein catabolic process, GO: 0019220∼regulation of phosphate metabolic process, GO: 0032268∼regulation of cellular protein metabolic process) (Table S9). The KEGG analysis of the identified targets of putative novel miRNAs associated with mitochondria of HEK293 showed the regulation of neurotrophin signaling, cell cycle, phosphatidyl inositol signaling system (Table S10).

Similarly, targets of putative novel miRNAs associated with mitochondria from HeLa cells were also analyzed. The GO term related to apoptosis, cell cycle, stress response (GO: 0042981∼regulation of apoptosis, GO: 0051726∼regulation of cell cycle cellular processes, GO: 0000079∼regulation of cyclin-dependent protein kinase activity, GO: 0001938∼positive regulation of endothelial cell proliferation) were the enriched clusters (Table S11). KEGG analysis of identified targets of putative novel miRNAs associated with mitochondria from HeLa showed the gene network involved in endocytosis, p53 signaling, adherence junction, dilated cardiomyopathy and cancer (Table S12).

### Validation of known miRNAs and Target mRNAs Associated with Human Mitochondria

**Figure 7:**
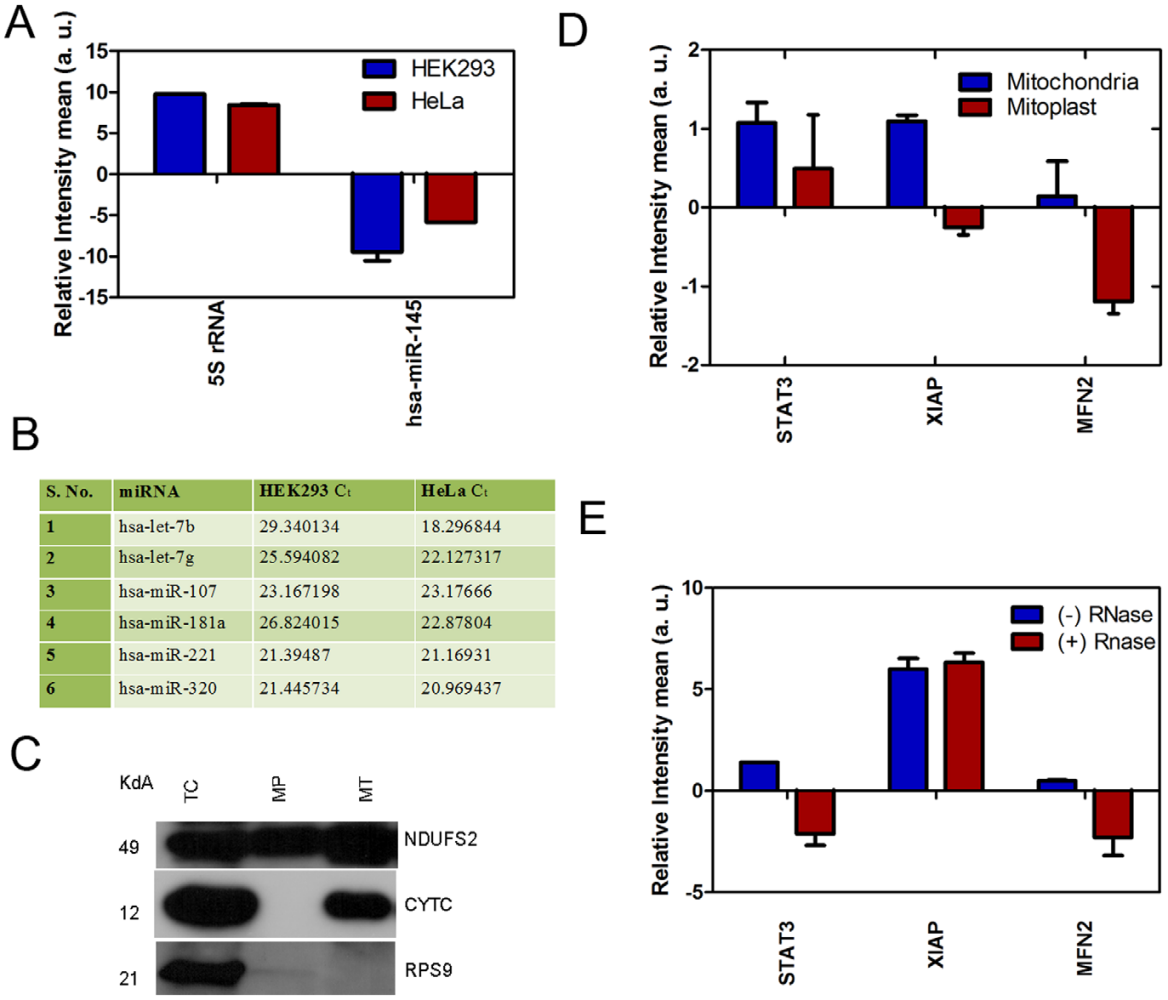
Validation of miRNAs and their targets associated with mitochondria. The enrichment of target mRNA of mitochondria-associated miRNAs with outer mitochondrial membrane were validated. (A) The association of 5S rRNA and negative control (hsa-miR-145) to mitochondria was analyzed. The relative enrichment of 5S rRNA and hsa-miR-145 was determined by qPCR as described in methods. (B) The miRNAs (frequency >1000) which were common in both the libraries were selected for validations by RT-qPCR. RNA was prepared from purified mitochondria and cDNA synthesized. The mean CT values of miRNAs (with CT <30 and distinct melt curve) from the mitochondria of both HEK293 and HeLa are listed. (C) The mitoplast was prepared from HEK293 as described in method section. The purity of mitoplast preparation was assessed by western blotting by probing with antibody against NDUFS2 (mitochondrial inner membrane protein) and cytochrome c (inter membrane space protein). TC: total cell; MP: mitoplast; MT: mitochondria. The targets of 3 miRNAs associated with mitochondria (let-7b: STAT3; hsa-miR-107: MFN2; hsa-miR-320a: XIAP) were determined by Starbase and validated by qPCR. (D) Validation of target mRNA associated with mitoplast as compared to mitochondria from HEK293. (E) Validation of target mRNA associated with RNase A treated mitochondria from HEK293.

The experiments were done to validate the association of miRNAs with mitochondria. The non specific association of miRNA was excluded by analyzing controls based on previous experiments. U6 snRNA and 5S rRNA was taken as endogenous and positive controls respectively due to their known association with mitochondria [29], [27]. We also observed high levels of U6 in our sRNA microarray analysis of mitochondrial RNA from HEK293 (data not published). Our microarray results also demonstrated that hsa-miR-145 was not detected in mitochondrial fraction whereas predominantly present in the total cell suggesting that it is not associated with mitochondria, hence was taken as negative control. The analysis by real time PCR showed that 5S rRNA was significantly associated whereas no association of hsa-miR-145 was observed in the RNA fraction isolated from purified mitochondria (Figure 7A). This experiment here as well as results from previous experiment clearly showed that specific miRNA associate with mitochondria and there is no non-specific association. The miRNAs with high count from both libraries (let-7b, let-7g, hsa-miR-107, hsa-miR-181a, hsa-miR-221 and hsa-miR-320a) were considered for the analysis for their association with mitochondria. The miRNAs assessed by RT-qPCR followed similar pattern of association (Figure 7B) supporting the sequencing results. Argonaute proteins bind to guide strand of mature miRNA and regulate the process of translation. The association of miRNA to mitochondria also suggests that Ago proteins may also be localized to mitochondria hence we analyzed the subcellular localization of Ago2/3 proteins. We observed that Ago2 colocalized with mitochondria (Figure S3) which was in consonance with earlier observations [27], [29]. Ago3 also colocalized with mitochondria whereas GFP vector (negative control) showed no co-localization with mitochondria.

We hypothesized that if these miRNAs and miRISC components are associated with mitochondria, the target mRNAs may also be associated to the outer surface of mitochondria. We analyzed the targets of 3 miRNAs (let-7b: STAT3; hsa-miR-107: MFN2; hsa-miR-320a: XIAP) by StarBase using intersection of 3 computational tools as described in materials and methods and checked their association with mitochondria. To check the association of miRNA/target mRNA with outer membrane the levels of target mRNAs were analyzed by qPCR in both mitochondria and mitoplast. The mitoplast preparation was checked prior to isolation of RNA by western blotting. The inner membrane localized protein, NDUFS2 was significantly enriched in mitoplast whereas, the inter membrane space protein cytochrome c was absent in mitoplast (Figure 7C). The levels of target mRNAs (STAT3, MFN2 and XIAP) of selected miRNAs (let-7b, hsa-miR-107, hsa-miR-320a) were significantly associated with mitochondria whereas the levels decreased significantly in mitoplast (Figure 7D). This was also confirmed by analyzing the association of mRNAs with mitochondria after RNase A treatment. RNase A treatment resulted in significant decrease in levels of target mRNAs (STAT3 and MFN2) whereas the levels of XIAP remained unchanged (Figure 7E). This may be due to association of XIAP mRNA with RNP complexes or localization site in mitochondria which may be not be accessible by RNase A for degradation. These evidences suggest that the subunits of RISC complex like Ago proteins localize to the outer membrane may bind to target mRNA and may serve as platform for assembly of site-specific regulation of mRNA turnover and protein levels.

## Discussion

The import of nuclear encoded tRNA and rRNA into the mitochondria has been demonstrated [17], [35]. This raised an interesting question of possible import of small non-coding RNAs including miRNA to the mitochondria, which may fine-tune the levels of target proteins associated with mitochondria and under various physiological conditions. In this study, we described the association of sRNAs including miRNA with mitochondria of two human cell lines HEK293 and HeLa through deep sequencing. The targets of identified known and putative novel miRNAs may regulate critical cellular pathways where mitochondria plays key role.

The deep sequencing of mitochondrial associated RNA confirmed the presence of diverse population of sRNAs: snRNA, srpRNA and snoRNA. The association of diverse population of sRNAs is intriguing and suggests either the role of mitochondria in their biogenesis or the role of sRNA in regulation of mitochondrial functions. The snRNA and snoRNA are transported back from cytoplasm to sub-nuclear regions [36]. It has been observed that snoRNA are processed in a similar manner like miRNA [36] and may perform functions similar to miRNA apart from its role in ribosomal biogenesis. The association of snoRNA with mitochondria needs to be further studied for their role in mitochondrial ribosome assembly, RNA modifications or miRNA like functions. The association of srpRNA also suggests that mitochondria may be involved in the translational of the mRNA encoding mitochondrial targeted proteins. These observations were further strengthened from the recent study describing the mitochondrial transcriptome where snRNA, snoRNA and srpRNA were observed to be associated with mitochondria [37]. Interestingly, we also observed piRNA associated with mitochondria from both HEK293 and HeLa cells. It has been observed earlier that piRNAs are generally expressed in germ line tissues but emerging evidences suggest the presence of piRNA in different somatic tissue [38]. The expression of piRNA was observed in 17 out of the 40 mouse somatic tissues and cell types. The piRNA expressed in somatic tissues showed characteristics of piRNA such as length distribution between 24 and 32 nt, 5′ uridine bias and clustering along the genome along with universal piRNA features. The association of piRNA with mitochondria of HEK293 and HeLa in this study suggests either mitochondria are involved in biogenesis of piRNA or postranscriptionally associates with mitochondria like miRNA. The role of mitochondria in biogenesis of piRNA has been further supported by recent studies. Two groups have clearly demonstrated the role of mitochondrial protein known as MitoPLD/zuc (Drosophila homologue) in piRNA biogenesis and piRNA mediated silencing both in fly and mouse germlines [23], [39]. This suggests that mitochondria may be an important player in piRNA pathways.

Interestingly, 2–5% sRNA reads was categorized to be miRNA supporting the recent evidence of existence of mitochondrial associated miRNA [26], [28], [29]. During our manuscript preparation, mitochondrial transcriptome was analyzed [37] which is in consonance with our study and observed that 3% of sRNAs showed similarity to miRNA. Interestingly, we observed that miRNA identified in recent studies [28], [29], [37] as well as microarray of mitochondrial RNA from our lab (data not published) was found to be widely represented in our deep sequencing results. This strongly suggests that our deep sequencing approach confirmed the validated results observed in the previous studies. The further analysis showed that the frequency of association of 35 miRNAs with mitochondria of HEK293 and HeLa were similar whereas majority of miRNAs were differentially associated in both cell lines. Similarly, 196 and 13 putative novel miRNAs were associated with mitochondria from HEK293 and HeLa respectively. These evidences suggest that association of miRNAs with mitochondria may be cell type and stimulus specific which needs further analysis using different cell lines and in different conditions. Interestingly, base biasness of miRNA (22 nt) showed preference for U at the first position in both HEK293 and HeLa suggesting mitochondrial associated miRNA may have high affinity to preferentially bind to MID domain of Ago2 and may be involved in binding with targets for gene regulation [40]. Further, it was observed that 4 known and 24 putative novel miRNA aligned to mitochondrial genome at different positions corresponding to 16S rRNA, tRNA and subunits of complex I. These miRNAs may be either mitochondrial genome encoded or nuclear encoded, may regulate mitochondrial transcripts involved in ribosome assembly and electron transport chain. This will require further experimental validations.

We identified the targets of miRNAs associated with mitochondria with high frequency count from both HEK293 and HeLa. Interestingly, GO terms related to RNA transcription were enriched. Ago2, core component of RISC complex involved in regulation and turnover of target mRNA has been reported to be colocalized with mitochondria [41]. Our results also showed the association of Ago2 and Ago3 with mitochondria (Figure S3). It has been well established that Ago proteins associates with miRNA [42]. Interestingly, Ago1–3 has recently been demonstrated to be redundantly associated with diverse population of RNA apart from miRNA including tRNA, vRNA, snoRNA, rRNA pseudogenes, snRNA, tiRNA, TSSa-RNA, PASRs, etc [43] which may be one of the reasons explaining the observed association of these RNAs to mitochondria. These sRNAs are known to regulate mRNA turnover and translation which again emphasizes the possible role of mitochondria in these processes. Similarly, it has been observed that mitochondria also associate with P bodies [44]. These P bodies are cytoplasmic granules that are linked to mRNA decay, mRNA storage and RNA silencing. In our study, we also observed that miRNAs and target mRNAs associated with mitochondria were enriched to the outer membrane of mitochondria. This is also supported by recent study of mitochondrial transcriptome where depletion of outer membrane of mitochondria results in selective loss of miRNAs [37] and other RNAs. These evidences strongly suggest that the outer membrane may assemble novel complexes by associating the proteins of RISC complexes, target mRNA to regulate site-specific turnover of mRNA and translation.

Analysis of putative novel miRNAs associated with mitochondria from HEK293 and HeLa showed enrichment of GO functions related to positive/negative regulation of RNA which has been discussed above that further supported our hypothesis. Interestingly, putative novel miRNAs identified in our study also showed enrichment of GO functions related to several signaling pathways important in cancer and metabolism where mitochondria plays crucial role. Apoptosis, cell cycle, kinase activity and proteins transport related pathways were enriched. Some of the known miRNA-target interactions have been demonstrated previously. hsa-miR-103/107 is highly associated with mitochondria (as observed in our study) which regulates cellular Acetyl-CoA and lipid levels [45]. Similarly hsa-miR-181 targets multiple Bcl-2 family members and regulates apoptosis [46]. These evidences suggest that miRNAs associate with mitochondria and regulates the turnover of mRNA and levels of protein that are implicated in mitochondrial related functions.

In conclusion, our study using subcellular fractionation and deep sequencing demonstrated the association of sRNAs including miRNA to mitochondria. The putative novel miRNAs needs further characterization for their function, association with mitochondria and translocation to other subcellular sites during different cellular processes. The association of miRNAs, their target mRNAs and Ago2/3 with mitochondria suggests that mitochondrial outer membrane may probably provide novel platform to assemble the miRNA/RISC complexes to regulate the subcellular site-specific protein levels. The further study in this direction will help to understand many unanticipated role of mitochondria and associated miRNA in different physiological and pathological conditions.

## Materials and Methods

### Cell Culture

HEK293 and HeLa cells were grown at 5% CO2, 37°C in Dulbecco's Modified Eagle's Medium (DMEM, Gibco, Invitrogen, USA) supplemented with 1% penicillin, streptomycin and amphotericin B (PSA) antibiotic mixture (Gibco, Invitrogen, USA) and 10% (v/v) heat-inactivated fetal bovine serum (Gibco, Invitrogen, USA). HEK293-MTRFP stable cell line was generated as described in Methods S1.

### Preparation and Analysis of Purity of Mitochondria

Mitochondria were prepared and purified from HEK293 and HeLa cells using Qproteome Mitochondria Isolation Kit (Qaigen, USA) as per manufacturer’s instructions with minor modifications. Briefly, 5×10^8^ cells were suspended in Lysis buffer incubated in ice for 10 min and centrifuged at 1000× g for 10 min at 4°C. The pellet was washed again with Lysis buffer and resuspended in Disruption Buffer, passed 10 times through 24 gauge needle to ensure complete cell disruption and centrifuged at 1000×g. The supernatant was centrifuged at 8000 rpm for 10 min at 4°C, to pellet down mitochondria. The mitochondria were washed and purified by adding on top of layers of Purification Buffer and Disruption Buffer. The solution was centrifuged at 13000 rpm for 15 min at 4°C. The mitochondrial ring at the interface of Purification Buffer/Disruption Buffer was collected and washed in Mitochondria Storage Buffer.

The purity of the mitochondrial fraction was assessed by western blotting as described earlier [47] with minor modifications. Briefly, the mitochondria and total cells were solubilized in solubilizing buffer (50 mM Bis-Tris (pH 7.0), 750 mM e-amino-caproic acid, 0.1% TritonX-100 and 10 ug/ml protease inhibitor). The concentration of total cellular and mitochondrial lysate was determined by Bradford assay, normalized and resolved on 12.5% SDS-PAGE. The proteins were electroblotted on PVDF membrane at 100 V for one hour. The membrane was blocked with 5% non-fat dried milk and 0.1% Tween-20 in PBS for 1 hour at room temperature. The membrane was incubated with rabbit polyclonal antibody against NDUFS2 (mitochondrial complex I subunit). After incubation membrane was washed three times with PBS-T (PBS containing 0.1% Tween 20) for 10 minutes each and incubated with a secondary antibody at room temperature for 1 hr. The membrane was again washed three times with PBS-T and signal visualized by using EZ-ECL chemiluminescence detection kit (Biological Industries, Israel) by exposing to X-ray film. The membrane was restriped and further probed with polyclonal antibody against RPS9 (ribosomal protein S9). All primary antibodies were used at 1∶1000 dilutions.

The nuclear contamination and mitochondrial enrichment were checked at RNA level by RT-PCR. RNA was isolated from mitochondria and first strand cDNA was synthesized using iScript™cDNA Synthesis Kit (Bio-Rad, USA) at 42°C for 1 hour, followed by inactivation of enzyme at 85°C for 5 min. The β-actin (Fwd: CTGGAACGGTGAAGGTGACA; Rev: AAGGGACTTCCTGTAACAATGCA) gene was taken as nuclear control whereas mitochondrial DNA encoded mRNA as positive control (ND4 (Fwd: ACAAGCTCCATCTGCCTACGA; Rev: GGCTGATTGAAGAGTATGCAATGA)) for PCR amplification. The reactions were performed at annealing temperature of 56°C.

### RNA Isolation from Purified Mitochondria

RNA was extracted from purified mitochondria and total cell using miRCURY™ RNA Isolation Kit according to manufacturer’s instructions (Exiqon, Germany). Briefly, the purified mitochondria were resuspended in RNA lysis buffer and incubated at room temperature in column for 5 min to allow binding. The column was washed thrice with washing buffer at 14000 rpm for 1 min. The RNA was eluted in nuclease free water from the column. Purity and concentration of RNA was determined using NanoPhotometer (Implen, Germany). The integrity of mitochondrial and total cellular RNA was checked with Agilent 2100 Bioanalyzer (Agilent Technologies, Inc., USA).

The cytosolic mRNA contamination was checked by RT-qPCR. The mitochondrial encoded mRNAs: ND4 (Fwd: ACAAGCTCCATCTGCCTACGA; Rev: GGCTGATTGAAGAGTATGCAATGA) and CYB (Fwd: AACCGCCTTTTCATCAATCG; Rev: AGCGGATGATTCAGCCATAATT); cytosolic mRNAs: TRIM4 (Fwd: CACCAGACTCACGCCATG; Rev: ACGAGATTACGCTGAGACTTAAG) and MITA (Fwd: CGCCTCATTGCCTACCAG; Rev: ACATCGTGGAGGTACTGGG) were relatively quantified taking RNP (Fwd: CCCCGTTCTCTGGGAACTC; Rev: TGTATGAGACCACTCTTTCCCATA) as endogenous control using SYBR® Premix Ex Taq™ (Takara, Japan) as per manufacturer’s instruction. Briefly, the initial denaturing was done at 95°C for 30 sec followed by 40 cycles of 95°C for 15 sec and 60°C for 60 sec. The specificity of each amplicon was checked by melt curve analysis. The reactions were performed using StepOnePlus™ (Applied Biosystem, USA). The reactions were performed in triplicates, ΔΔC_T_ calculated and relative association with standard error was plotted using Graph Pad PRISM 5.0.

### Isolation of Mitoplast

The mitoplast was prepared from purified mitochondria as described earlier [48] with minor modifications. The mitochondria (protein concentration of 1.0 mg/ml) were incubated in 10 mM KH_2_PO_4_ with 2.7 mg/ml digitonin for 20 min on ice. The solution was centrifuged at 10,000 g for 10 min at 4°C. The pellet was washed twice with PBS. The quality of preparation was confirmed by analyzing the presence/absence of cytochrome c by western blotting as described earlier.

### RNase A Treatment

The mitochondria were treated with RNase A to check the association of miRNAs and mRNA with outer surface of mitochondria as described previously [28]. The purified mitochondrial pellet was resuspended in 20 µl mitochondrial storage buffer and mixed with 300 µl buffer P1 (QIAprep Spin Miniprep Kit, Qiagen, USA) containing 40 µg/ml RNase A and incubated at 37°C for 1 hr. The reaction was stopped by adding Proteinase K (100 mg/ml) resuspended in PBS. RNase A treated mitochondria were pelleted down at 8000 rpm for 10 min and washed twice in mitochondrial storage buffer prior to RNA isolation.

### sRNA Library Preparation and Sequencing

RNA library preparation and sequencing was performed by BGI (Beijing Genomics Institute, Shenzhen, China**)**. Briefly, sRNA (18 to 30 nt) was gel purified and ligated to the 3′ and 5′ adaptor. The ligated products were used for cDNA synthesis, followed by acrylamide gel purification and PCR amplification for 14 cycles of 98°C for 10 sec and 72°C for 15 sec to generate sRNA libraries [30]. The library (1 µl) was loaded on an Agilent Technologies 2100 Bioanalyzer to check size, purity, and concentration. Libraries were sequenced on an Illumina HiSeq 2000 (Illumina, USA). Sequencing data was processed with the Illumina pipeline v1.3.2.

### Bioinformatics Analysis of sRNA Libraries from Mitochondria

All the 50 nt sequence tags generated from Illumina HiSeq 2000 went through data cleaning process which included getting rid of low quality tags. The clean tags lengths were then summarized. The clean tags were also mapped to the UCSC hg19 human assembly using SOAP (Short Oligonucleotide Alignment Program) [49] with the following options: soap -v 0 -r 2 -s 7 -p 7 -a. Sequences with perfect match or one mismatch were retained for further analysis. The presence of known miRNAs was analyzed by aligning clean tags to pre-miRNAs (1424), mature miRNAs (1539) and miRNA* (194) of miRBase 17.0 [50]. The other populations of non coding sRNAs (rRNA, scRNA, snoRNA, snRNA, tRNA and piRNA) were all identified by aligning the sequences to Genbank [51] and Rfam (www.sanger.ac.uk/resources/databases/rfam.html) using following program and parameters: blastall -p blastn -F F -e 0.01. The repeat associated RNA and piRNA were identified using tag2repeat and tag2piRNA respectively at BGI. sRNA tags were also aligned to exons and introns of mRNA to find the degraded fragments of mRNA. All annotations were summarized using tag2annotation software in the following order of preference: rRNA (Genbank > Rfam) > known miRNA > repeat > exon > intron.

### Analysis of Differential Association of miRNAs with Mitochondria of HEK293 and HeLa

To compare the differential association of miRNAs with mitochondria from HEK293 and HeLa, the miRNAs associated with mitochondria were normalized to obtain the level of association in terms of transcripts per million. If the normalized association value of a given miRNA is zero, its association value was modified to 0.01. Then, the fold-change and P-value were calculated from the normalized association using the following equations.
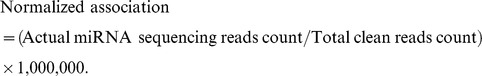


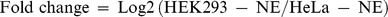



P-value was calculated using following equation as described previously [52].
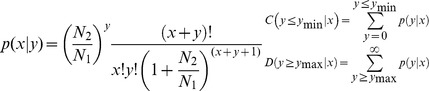



The N1 and x represent total count of clean reads and normalized association level of a given miRNA in HeLa sRNA library, respectively. The N2 and y represent total count of clean reads and normalized association level of a given miRNA in HEK293 sRNA library, respectively.

### Prediction of Putative Novel miRNA

All unannotated sRNA tags that aligned to UCSC hg19 were employed to screen putative novel miRNA using Mireap [http://sourceforge.net/projects/mireap/] algorithm under these parameter settings : miRNA sequence length (18–26 nt); miRNA reference sequence length (20–24 nt); Minimal depth of Drosha/Dicer cutting site (3); Maximal copy number of miRNA on reference (20); Maximal free energy allowed for a miRNA precursor (−18 kcal/mol); Maximal space between miRNA and miRNA* (35); Minimal base pairs of miRNA and miRNA* (14); Maximal bulge of miRNA and miRNA* (4); Maximal asymmetry of miRNA/miRNA* duplex (5); Flank sequence length of miRNA precursor (10). The base bias on the first position and different position was also determined.

### Clustering of miRNA Targets

The miRNAs associated with mitochondria were divided in two classes based on the number of reads: Class-I (count >5000) and Class II (count <5000). The targets were analyzed by using StarBase, a database that has been developed based on experimentally verified miRNA target interactions by CLIP-Seq (HITS-CLIP, PAR-CLIP) and degradome sequencing (Degradome-Seq, PARE) data [34]. Given that miRNA targets prediction tools give high number of false positive, only the target identified by three independent tools (PicTar, miRanda and TargetScan) and overlapped with CLIP-Seq data were taken into further consideration. The targets of putative novel miRNAs were also identified in similar way.

To characterize the biological processes regulated by targets of identified miRNAs associated with mitochondria, we used Gene Ontology (GO) and the functional annotation clustering feature of DAVID. This tool measures the similarities among related GO terms based on the extent of their associated genes and assembles the similar and redundant GO terms into annotation clusters. Fisher Exact p-value is assigned to each GO term that represents the degree of enrichment of the GO term in the input gene list. Finally, each cluster is given enrichment score to measure the biological significance. The resulting clusters were further curated to keep only GO terms with p-values >0.05. The genes with FDR≤0.05 were considered as significantly enriched in target gene candidates and were mapped in relevant signaling pathways in KEGG database [53].

### Alignment of sRNAs to Mitochondrial Genome

The clean reads from both the libraries were aligned to human mitochondrial genome NC_012920 [11] through MapMi version 1.0.4.02 (EMBL) (www.ebi.ac.uk/enright-srv/MapMi) and SOAP options: -v 0 -r 2 -s 7 -p 7–a. The other populations of non-coding sRNAs (rRNA, scRNA, snoRNA, snRNA, tRNA and piRNA) were all identified by aligning to Rfam and GenBank. The alignment of putative novel miRNAs was also done by BLASTN version 2.2.20 [54] to investigate their matching sequence in mitochondrial genome.

### Validation of miRNAs and their Targets Associated with Mitochondria

RNA was isolated from purified mitochondria as described above and cDNA were synthesized using Universal cDNA synthesis Kit (Exiqon, Denmark) at 42°C for 1 hr, followed by heat inactivation of enzyme at 85°C for 5 minutes. The levels of following miRNAs were determined from 10 ng cDNA using U6 snRNA (Accession number: X59362) and 5 S rRNA (NCBI Accession no: V00589) as endogenous control using miRCURY LNA™ Universal RT microRNA PCR, SYBR Green master mix (Exiqon, Denmark):

hsa-let-7b (UGAGGUAGUAGGUUGUGUGGUU);

hsa-let-7g (UGAGGUAGUAGUUUGUACAGUU);

hsa-miR-107 (AGCAGCAUUGUACAGGGCUAUCA);

hsa-miR-181a (AACAUUCAACGCUGUCGGUGAGU);

hsa-miR-221 (AGCUACAUUGUCUGCUGGGUUUC);

hsa-miR-320a (AAAAGCUGGGUUGAGAGGGCGA);

hsa-miR-145 (GUCCAGUUUUCCCAGGAAUCCCU).

The initial denaturing was done at 95°C for 10 min followed by 40 cycles of 95°C for 15 sec and 60°C for 60 sec followed by melt curve analysis to check the specificity.

The enrichment of target mRNAs of mitochondria-associated miRNAs was determined by qPCR using following gene specific primers: XIAP (Fwd: GCACGGATCTTTACTTTTGGG, Rev: GGGTCTTCACTGGGCTTC); MFN2 Fwd: ATGTGGCCCAACTCTAAGTG, Rev: CACAAACACATCAGCATCCAG); STAT3 (Fwd: TTCTGGGCACAAACACAAAAG, Rev: TCAGTCACAATCAGGGAAGC) as described previously.

## Supporting Information

Figure S1 **The genomic mapping of mitochondria-associated sRNAs.** The clean sequence reads were mapped onto human reference genome (UCSC hg19) to determine the genomic locations of small RNA (sRNA) using SOAP. (A) The number of sRNA tags associated with mitochondria of HEK293 mapped at specific chromosomal position. (B) The number of sRNA tags associated with mitochondria of HeLa mapped at specific chromosomal position. The area above 0 is the number of small RNAs (sRNAs) on sense strand of chromosome, shown in blue whereas area below 0 is the number of sRNAs on the antisense strand of the chromosome, shown in red.
(TIF)

Figure S2 **Classification of repeat associated reads from mitochondria-associated sRNA libraries.** The total sequence reads classified as repeat associated elements were further categorized to determine the levels of each repeat associated RNAs in graphical format. (A) Detailed clustering of repeat-associated RNAs from mitochondria-associated sRNA library of HEK293. (B) Detailed clustering of repeat-associated RNAs from mitochondria-associated sRNA library of HeLa.
(TIF)

Figure S3 **Association of human Argonaute with mitochondria.** Argonaute proteins co-localizes with mitochondria. HEK293-MTRFP cells were transfected with pEGFPC1-Ago2, pEGFPC1-Ago3 and pAcGFP-N1. After 24 hrs of transfection, cells were stained with Hoechst and analyzed by confocal microscopy as described in Methods S1.
(TIF)

Table S1 **Annotations of various classes of sRNA associated with mitochondria of HEK293.** The total clean sequences obtained from the sRNA libraries were subjected to a series of sequence similarity searches using specific databases (rRNAs, tRNAs, sn/snoRNAs, miRNAs, other non-coding RNAs). The sequences that did not match with any known sequence were categorized as unannotated sequences. All annotations were summarized using tag2annotations software. An overview of sRNAs associated with mitochondria of HEK293. ^1^ type of sRNA, ^2^ total number of unique sequences belonging to each category,^ 3^ percentage of unique sequences belonging to each category,^ 4^ total number of all sequences belonging to each category,^ 5^ percentage of total sequences belonging to each category.
(XLS)

Table S2 **Annotations of various classes of sRNA associated with mitochondria of HeLa.** An overview of sRNAs associated with mitochondria of HeLa. ^1^, ^2^, ^3^, ^4^, ^5^ same as table S1.
(XLS)

Table S3 **Pattern of miRNAs associated with mitochondria of HEK293 and HeLa.** The miRNAs associated with mitochondria from both cell lines and their respective frequency count. ^1^ name of miRNA according to miRBase 17.0, ^2^ and ^3^ total sequences reads that matched to particular miRNA from mitochondria-associated sRNA library of HEK293 and HeLa respectively.
(XLS)

Table S4 **Putative novel miRNAs associated with mitochondria of HEK293 and HeLa.** Features of putative novel miRNAs associated with mitochondria of HEK293 and HeLa as determined by miReap. ^1^ identification code assigned to each putative novel miRNA, ^2^ genomic location of each putative novel miRNA, ^3^ orientation of putative novel miRNA on chromosome (+/−), ^4^ MFE energy score (<−18 kcal/mol) of each miRNA, ^5^ number of sequence reads matched from the library, ^6^ sequence of putative novel miRNA.
(XLS)

Table S5 **The GO term of predicted targets of miRNAs associated with mitochondria (HEK293 and HeLa) belonging to high frequency count category (count >5000).** The targets of miRNAs associated with mitochondria (HEK293 and HeLa) belonging to high frequency count category (count >5000) were determined by StarBase and clustered into GO terms using the DAVID gene annotation tool. ^1^ Number of cluster and enrichment score (ES) >1.05, ^2^ The gene annotation term, ^3^ The number of target genes which belonged to GO cluster, ^4^ Fisher exact p-value representing the degree of enrichment of the GO term, ^5^ Benjamini correction value for each category.
(XLS)

Table S6 **KEGG pathways enriched for targets of miRNAs associated with mitochondria (HEK293 and HeLa) belonging to high frequency count category (count >5000).** The targets of miRNAs associated with mitochondria (HEK293 and HeLa) belonging to high frequency count category (count >5000) were determined by StarBase and clustered into KEGG pathways using the DAVID gene annotation tool. ^1^ KEGG pathway and its ID, ^2^ the number of target genes, which belong to the pathway, ^3^ Fisher Exact p-value representing the degree of enrichment, ^4^ Benjamini correction value for each category.
(XLS)

Table S7 **The GO term of predicted targets of miRNAs associated with mitochondria (HEK293 and HeLa) belonging to low frequency count category (count <5000).** The targets of miRNAs associated with mitochondria (HEK293 and HeLa) belonging to low frequency count category (count <5000) were determined by StarBase and clustered into GO term using the DAVID gene annotation tool. ^1^, ^2^, ^3^, ^4^,^ 5^ same as table S5.
(XLS)

Table S8 **KEGG pathways enriched for targets of miRNAs associated with mitochondria (HEK293 and HeLa) belonging to low frequency count category (<5000).** The targets of miRNAs associated with mitochondria (HEK293 and HeLa) belonging to low frequency count category (count <5000) from were determined by StarBase and clustered into KEGG pathways using the DAVID gene annotation tool. ^1^, ^2^, ^3^, ^4^ same as table S6.
(XLS)

Table S9 **The GO term of predicted targets of putative novel miRNAs associated with mitochondria of HEK293.** The targets of novel miRNAs associated with mitochondria of HEK293 were determined by StarBase and clustered into GO terms using the DAVID gene annotation tool. ^1^, ^2^, ^3^, ^4^, ^5^ same as table S5.
(XLS)

Table S10 **KEGG pathways enriched for targets of putative novel miRNAs associated with mitochondria of HEK293.** The targets of putative novel miRNAs associated with mitochondria of HEK293 were determined by StarBase and clustered into KEGG pathways using the DAVID gene annotation tool. ^1^, ^2^, ^3^, ^4^ same as table S6.
(XLS)

Table S11 **The GO term of predicted targets of putative novel miRNAs associated with mitochondria of HeLa.** The targets of putative novel miRNAs associated with mitochondria of HeLa were determined by StarBase and clustered into GO term using the DAVID gene annotation tool. ^1^, ^2^, ^3^, ^4^, ^5^ same as table S5.
(XLS)

Table S12 **KEGG pathways enriched for targets of putative novel miRNAs associated with mitochondria of HeLa.** The targets of putative novel miRNAs associated with mitochondria of HeLa were determined by StarBase and clustered into KEGG pathways using the DAVID gene annotation tool. ^1^, ^2^, ^3^, ^4^ same as table S6.
(XLS)

Methods S1 **Supplementary Methods.**
(DOC)
