# Supplementary figures and images for: Systematic Analysis of Small RNAs Associated with Human Mitochondria by Deep Sequencing: Detailed Analysis of Mitochondrial Associated miRNA
Source: PLoS One. 2012 Sep 11;7(9):e44873. doi: 10.1371/journal.pone.0044873 (PMC3439422; doi:10.1371/journal.pone.0044873)

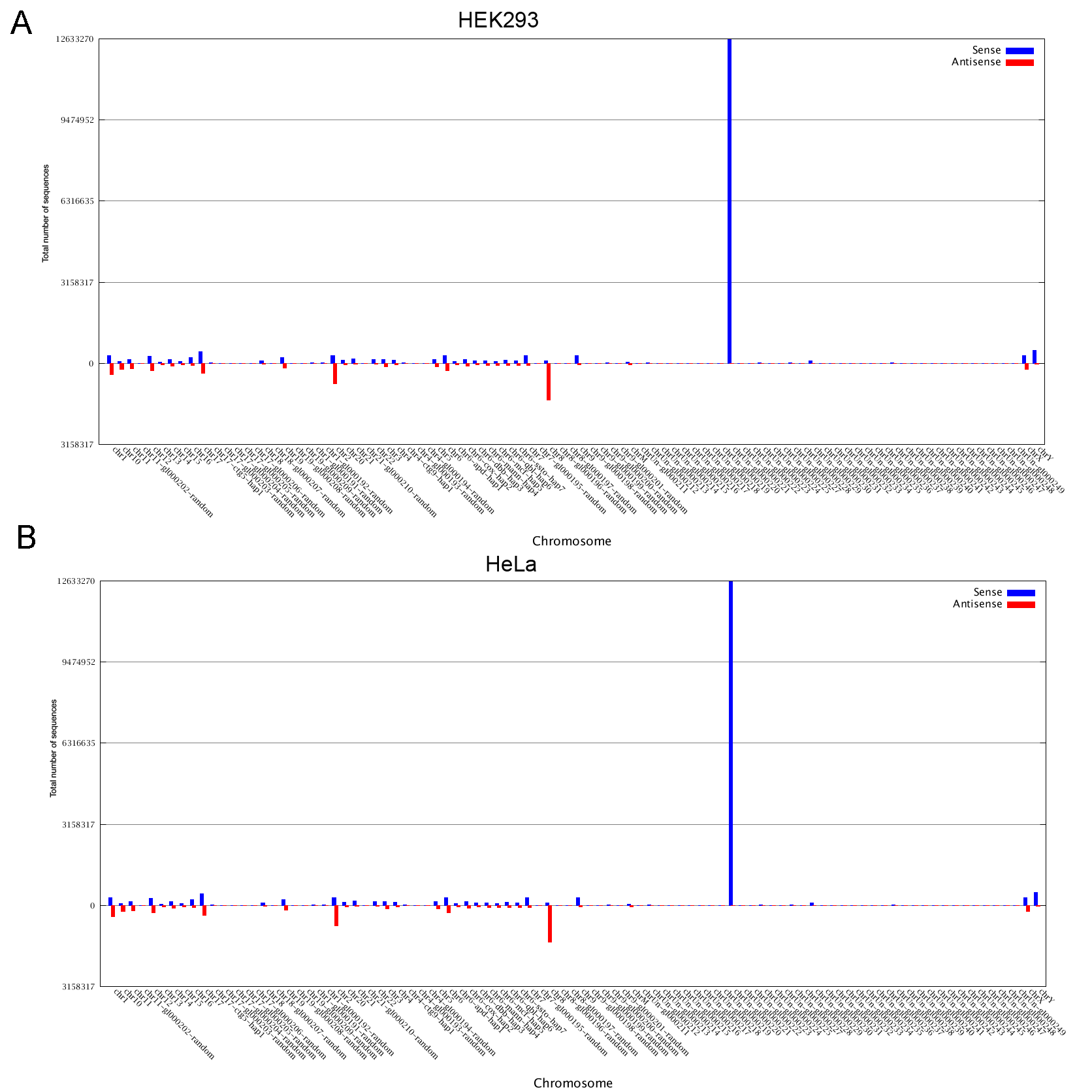

Supplement: Figure S1 — The genomic mapping of mitochondria-associated sRNAs. The clean sequence reads were mapped onto human reference genome (UCSC hg19) to determine the genomic locations of small RNA (sRNA) using SOAP. (A) The number of sRNA tags associated with mitochondria of HEK293 mapped at specific chromosomal position. (B) The number of sRNA tags associated with mitochondria of HeLa mapped at specific chromosomal position. The area above 0 is the number of small RNAs (sRNAs) on sense strand of chromosome, shown in blue whereas area below 0 is the number of sRNAs on the antisense strand of the chromosome, shown in red. (TIF) [file pone.0044873.s001.tif]

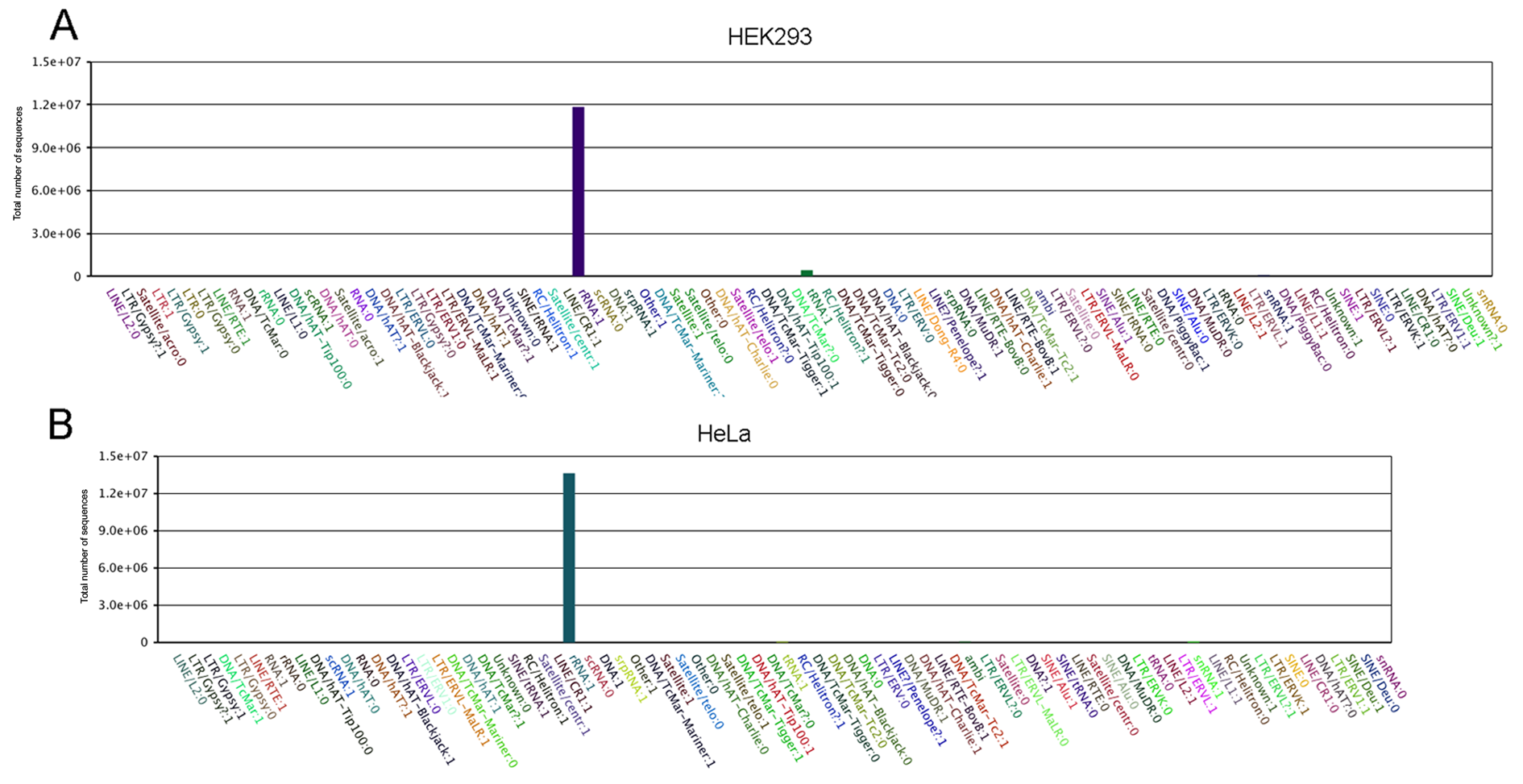

Supplement: Figure S2 — Classification of repeat associated reads from mitochondria-associated sRNA libraries. The total sequence reads classified as repeat associated elements were further categorized to determine the levels of each repeat associated RNAs in graphical format. (A) Detailed clustering of repeat-associated RNAs from mitochondria-associated sRNA library of HEK293. (B) Detailed clustering of repeat-associated RNAs from mitochondria-associated sRNA library of HeLa. (TIF) [file pone.0044873.s002.tif]

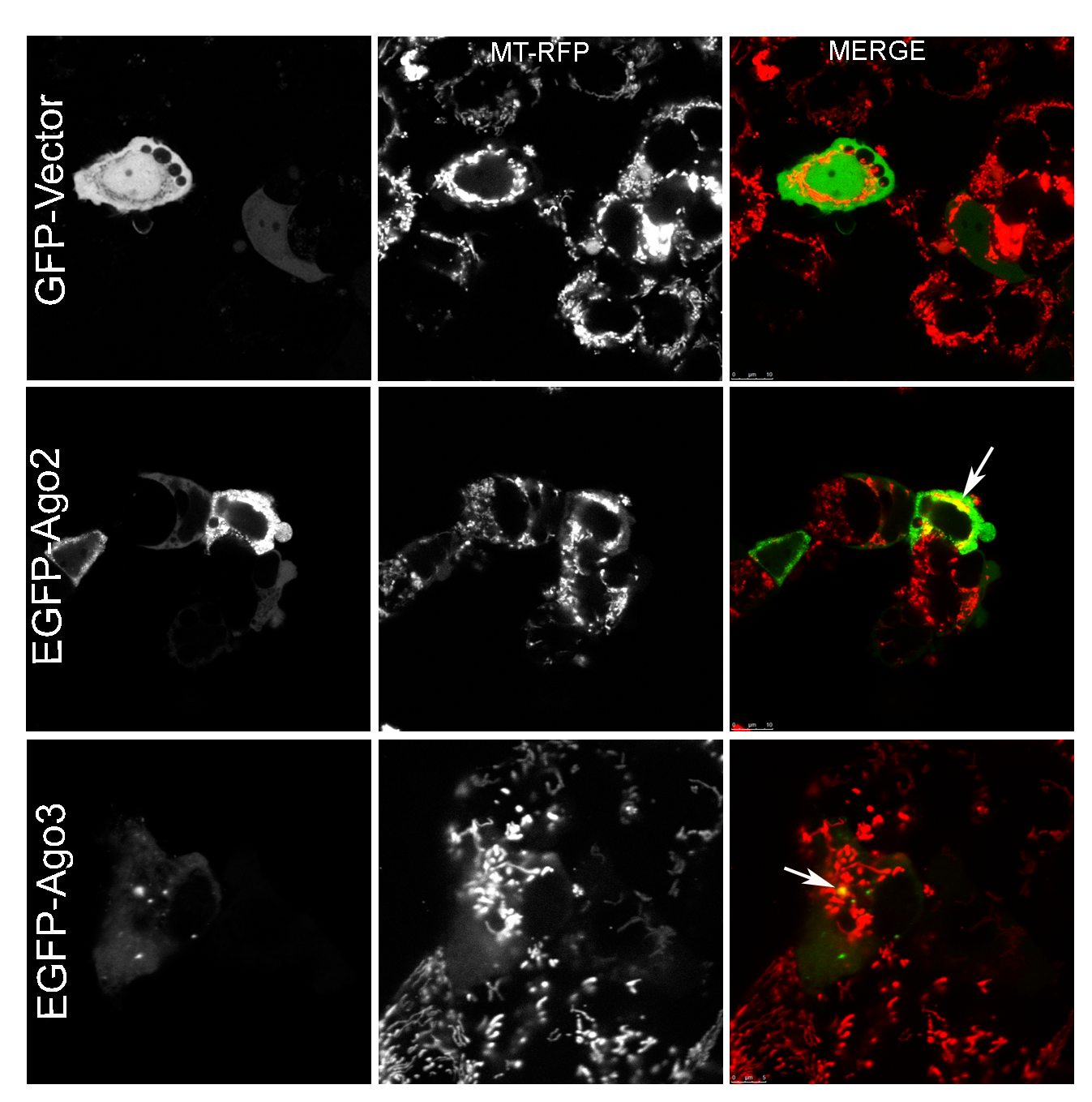

Supplement: Figure S3 — Association of human Argonaute with mitochondria. Argonaute proteins co-localizes with mitochondria. HEK293-MTRFP cells were transfected with pEGFPC1-Ago2, pEGFPC1-Ago3 and pAcGFP-N1. After 24 hrs of transfection, cells were stained with Hoechst and analyzed by confocal microscopy as described in Methods S1. (TIF) [file pone.0044873.s003.tif]
