# Supplementary material for: Systematic Analysis of Small RNAs Associated with Human Mitochondria by Deep Sequencing: Detailed Analysis of Mitochondrial Associated miRNA
Source: PLoS One. 2012 Sep 11;7(9):e44873. doi: 10.1371/journal.pone.0044873 (PMC3439422; doi:10.1371/journal.pone.0044873)
Supplement: Methods S1 — Supplementary Methods. (DOC) [file pone.0044873.s016.doc]

**SUPPLEMENTARY METHODS**

**Generation of stable cell lines**

To generate HEK293-MTRFP stable cell line, 1.5×10^5^ cells of HEK293 were plated in 24 well plate. After overnight incubation, mitochondrial targeted red fluorescent protein vector (pHcRed1-Mito) (Clonetech, USA) was transfected using calcium phosphate transfection method . Media was changed with DMEM supplemented with G418 (500 μg/ml) after 24 hrs of transfection and every alternate day until stable clones were clearly visible. The stable cells were harvested from 24 well plate and transferred to 96 well plate to obtain single clone using serial dilution method. The single clones were transferred and maintained in 12 well plates. After incubation for 7 days, the cells were transferred to 25 cm^2^ culture flask and maintained in DMEM supplemented with 200 μg/ml of G418.

**Confocal microscopy**

The co-localization of Ago2 and Ago3 with mitochondria was analyzed by confocal microscopy. HEK293-MTRFP cells were plated in 24 well optical bottom plate (Greiner Bio, USA) with cell density of 1.5×10^5^ per well. After 12 hrs of plating, the cells were transfected with pEGFPC1-Ago2, pEGFPC1-Ago3 and pAcGFP-N1 as described earlier. After 24 hrs of transfection, nucleus was stained with Hoechst. The cells were analyzed by Leica TCS SP5-II confocal microscope (Leica Microsystems, Germany) by sequential imaging of Hoechst, GFP and RFP fusion proteins using 63X objective with 3.0 X zoom.

**References**

1. Sano E, Shono S, Tashiro K, Konishi H, Yamauchi E, et al. (2008) Novel tyrosine phosphorylated and cardiolipin-binding protein CLPABP functions as mitochondrial RNA granule. Biochimica et biophysica acta 1783: 1036-1047.
